# Nucleic acid functionalized extracellular vesicles as promising therapeutic systems for nanomedicine

**DOI:** 10.20517/evcna.2021.21

**Published:** 2022-02-22

**Authors:** Chunping Liu, Dongyue He, Huan Cen, Huiqi Chen, Longmei Li, Guangning Nie, Zixue Zhong, Qingfeng He, Xiaofei Yang, Sien Guo, Lei Wang, Zhijin Fan

**Affiliations:** ^1^State Key Laboratory of Dampness Syndrome of Chinese Medicine, The Second Affiliated Hospital of Guangzhou University of Chinese Medicine, Guangzhou 510080, Guangdong, China.; ^2^State Key Laboratory of Quality Research in Chinese Medicine, Institute of Chinese Medical Sciences, University of Macau, Macau 999078, China.; ^3^Molecular Diagnosis and Treatment Center for Infectious Diseases, Dermatology Hospital, Southern Medical University, Guangzhou 510091, Guangdong, China.; ^4^School of Medicine, South China University of Technology, Guangzhou 510006, Guangdong, China.

**Keywords:** Extracellular vesicles, nucleic acid, nanomedicine, mRNA vaccine, aptamer

## Abstract

Extracellular vesicles (EVs), as natural carriers, are regarded as a new star in nanomedicine due to their excellent biocompatibility, fascinating physicochemical properties, and unique biological regulatory functions. However, there are still some challenges to using natural EVs, including poor targeting ability and the clearance from circulation, which may limit their further development and clinical use. Nucleic acid has the functions of programmability, targeting, gene therapy, and immune regulation. Owing to the engineering design and modification by integrating functional nucleic acid, EVs offer excellent performances as a therapeutic system *in vivo*. This review briefly introduces the function and mechanism of nucleic acid in the diagnosis and treatment of diseases. Then, the strategies of nucleic acid-functionalized EVs are summarized and the latest progress of nucleic acid-functionalized EVs in nanomedicine is highlighted. Finally, the challenges and prospects of nucleic acid-functionalized EVs as a promising diagnostic system are proposed.

## INTRODUCTION

Extracellular vesicles (EVs) are natural nano-carriers produced by living cells for intercellular communication^[1-3]^. EVs can be classified as exosomes, microvesicles, or apoptotic bodies according to their biogenesis type and particle size^[4]^. Exosomes and microvesicles are the most widely studied, thus “EV” is commonly used to refer to these two subgroups^[5]^. Exosomes, with particle sizes ranging from 30 to 150 nm, are formed when multivesicular bodies fuse with the cell membrane and release the vesicles inside^[6]^. Microvesicles, with particle sizes of 50-1000 nm, are formed by cell membrane bubbling^[6]^. Due to the limitation of the separation method, exosomes and microvesicles are difficult to separate in the range of 30-200 nm, which are commonly referred to collectively as small EVs. The EVs summarized in this paper mainly refer to small EVs, including exosomes and microvesicles. Due to their good biocompatibility, low immunogenicity, excellent extensibility, and unique biological regulatory function, EVs have attracted wide attention in the field of nanomedicine and are considered as a new star in nanomedicine^[7-9]^.

EVs have been developed as a delivery carrier of drugs or contrast agents, showing great application potential in the field of disease diagnosis and treatment^[5,8,9]^. However, native EVs have difficulty meeting the functional requirements of the complex physiological environment; therefore, necessary engineering design and modification can significantly improve the performance of EVs as a therapeutic system. As biological macromolecules, nucleic acid has unique biological functions and has been widely used in the field of nanomedicine^[10-13]^. RNA interference, antisense oligonucleotides, and cluster regularly spaced short palindromic repeats-associated protein 9 (CRISPR/Cas9) system can downregulate, enhance, or correct gene expression and have wide application potential in gene therapy research^[14-19]^. Nevertheless, these promising therapies are severely limited by inefficient biological distribution and sensitivity to degradation. The development of intracellular delivery carriers can effectively overcome the above limitations of nucleic acid therapy. EVs are natural carriers of information, matter, and energy exchange between cells, involving molecular transport between cells. Functional genetic components such as DNA, mRNA, and ncRNA loaded by EVs can be transported to target cells to perform the function of gene expression regulation. This suggests that EVs are a good nucleic acid delivery carrier. The combination of nucleic acid and EVs makes up for their shortcomings and is expected to provide a promising diagnosis and treatment system for nanomedicine. In addition, nucleic acids also have targeting (aptamer), programmability, drug loading, and immunomodulatory functions^[20]^, which will greatly improve the application prospects of EVs.

**Scheme 1:**
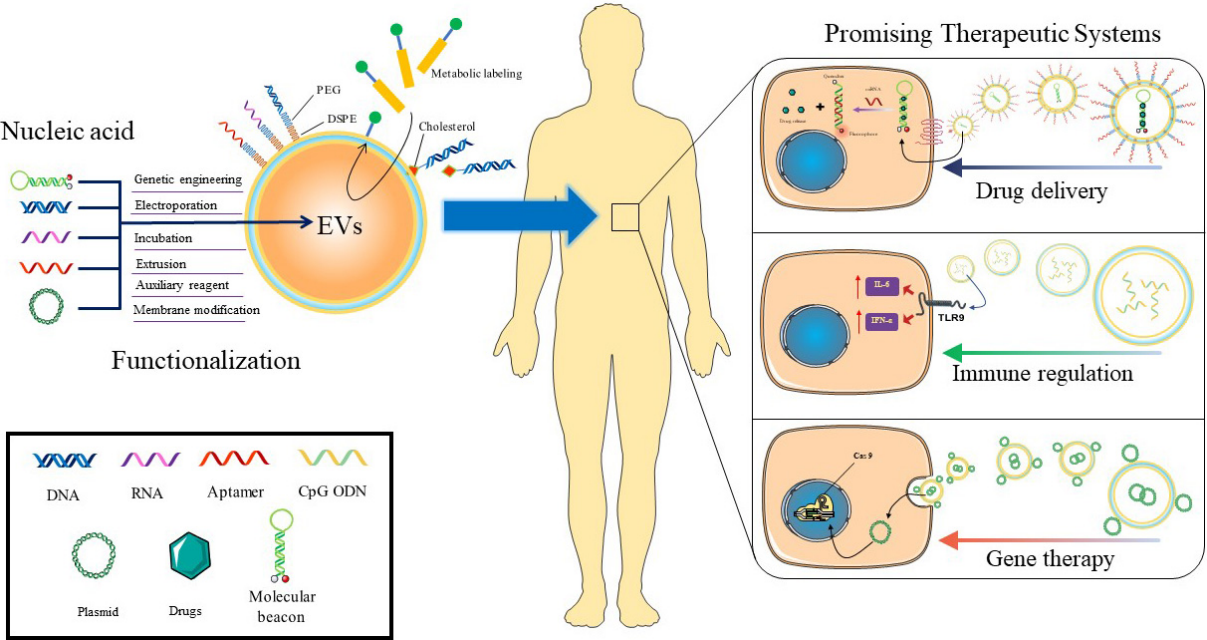
Nucleic acid-functionalized EVs are promising therapeutic systems for nanomedicine. As a functional macromolecule, nucleic acid has the capabilities of targeting, self-assembly, drug loading, gene editing, and immune regulation. By combining nucleic acid with EVs, EVs acquire the functional properties of nucleic acid, thus showing unprecedented application potential in drug delivery, immune regulation, and gene therapy, which are expected to provide a promising therapeutic system. EVs: Extracellular vesicles.

This review briefly summarizes the function and mechanism of nucleic acid in diagnosis and treatment and preliminarily clarifies the necessity and advantages of nucleic acid-functionalized EVs. This review provides a basic understanding of this field by highlighting the engineering strategies and representative progress (Scheme 1). Finally, the challenges and future development of nucleic acid-functionalized EVs are proposed.

## FUNCTION AND MECHANISM OF NUCLEIC ACID IN NANOMEDICINE

**Figure 1:**
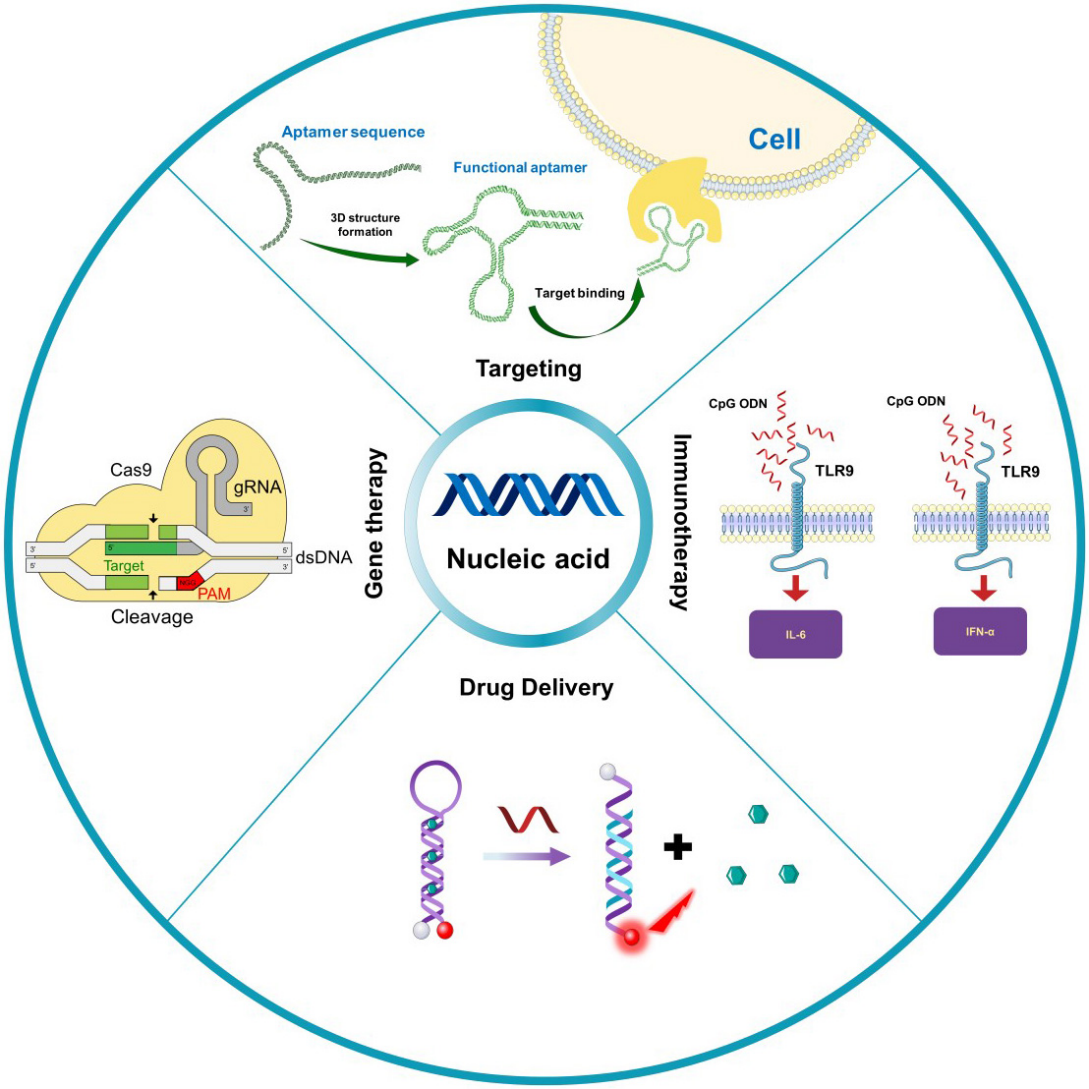
Function and mechanism of nucleic acid in nanomedicine. Nucleic acid has the characteristics of targeting (aptamer), immune regulation (CpG OND), drug delivery (molecular beacon or DNA origami), and gene editing (CRISPR/Cas9). CRISPR/Cas9: Cluster regularly spaced short palindromic repeats-associated protein 9.

As biological macromolecules, nucleic acid has unique biological functions and has been widely used in the field of nanomedicine^[21-23]^. Among them, the most common functions are targeting, programming, gene expression regulation, and immune regulation. This section briefly introduces the functions and mechanisms of nucleic acid [Figure 1]. Related studies on the use of nucleic acid in the biomedical field can also be found in earlier literature^[24,25]^.

### Targeting ability

Aptamers are oligonucleotide sequences with specific affinity activity screened by the systematic evolution of ligands by exponential enrichment technique. The obtained oligonucleotide sequences with specific recognition and affinity for proteins, bacteria, cells, and other target molecules are also called chemical antibodies^[26,27]^. Aptamers can be used as drugs themselves or combined with drugs, siRNA, and nanoparticles to form targeted drug delivery systems, which can target specific tumor cells, reduce toxicity to normal cells, significantly reduce drug dosage, and improve efficacy^[28-31]^. Aptamers have become valuable affinity probes in biochemistry research, disease diagnosis, and treatment. Recently, Wang *et al.*^[32]^ developed a DNA adapter with excellent targeting properties and unique functional versatility that can be used for biomarker detection, medical molecular imaging, and therapeutic targeted drug delivery. In another study, Liu *et al.*^[33]^ developed a fluorescent probe based on DNA aptamer for specific molecular typing of mammary neoplasms. Aptamers for new targets are being screened, providing new options for targeted therapy.

The aptamer, as a specific recognition element, has the advantages of simple synthesis, easy modification, biodegradability, and low toxicity, and it has aroused wide attention in both basic and clinical research^[28]^. In particular, some aptamers for surface biomarkers of cancer have been screened out and used in the design of targeted delivery systems for cancer^[34-36]^. With the development of technology, aptamers can be modified by various functional groups, which will further expand the application prospects of aptamers.

### Drug delivery carriers

Nucleic acids have the property of self-assembly, and they can be assembled into a double helix structure through complementary pairs of bases, or complex structures such as G-quadruplets can be constructed through complementary pairs of bases^[37,38]^. DNA origami technology uses the folding and self-assembly of nucleic acids such as DNA and RNA to form complex structures^[39,40]^. DNA origami technology can synthesize homogeneous nanostructures with sizes between 50 and 400 nm that can be used as drug delivery materials to enhance drug delivery and survival in malignant environments^[40-43]^. DNA origami technology can also design dynamic, multi-stimulus responsive nanostructures to achieve controlled release of drugs^[44,45]^. Jiang *et al.*^[46]^ used DNA origami to deliver adriamycin into the body. They found that drug-loaded DNA triangle origami showed a strong tumor treatment effect, and no systemic side effects were observed when treating human MDA-MB-231 breast tumor cells^[47]^. As an effective and biocompatible drug carrier, DNA origami has great potential in tumor therapy^[41,48-53]^.

In addition to DNA origami, there are other types of nucleic acid drug carriers. Molecular beacons are fluorescently labeled stem-loop oligonucleotide chains capable of loading and transporting doxorubicin^[54]^. The advantage of molecular beacons as drug carriers is that drug release requires conditions to trigger the destruction of nucleic acid secondary structure, and the drug release process can be monitored in real time by fluorescence signal. For example, Ma *et al.*^[55]^ reported a drug delivery system based on molecular beacon for detecting telomerase activity and telomerase triggered drug release in living cells. This provides a feasible strategy for conditionally controlled release and treatment monitoring. DNA hydrogel is a new kind of important DNA material, which is a three-dimensional polymer network constructed by DNA as a structural element^[56]^. It has been used extensively to develop drug delivery systems (DDS) because of its advantages of high water content, large drug loading space, and good biocompatibility^[57]^. To sum up, the nucleic acid drug carrier has the advantage of being programmable, showing great application potential in the construction of DDS.

However, naked nucleic acid nanostructures have relatively high electrical charges, which may influence their behavior in blood circulation and scavenging. Additionally, the DNA nanostructure has a potential immune risk, being it easy to trigger the body’s inflammatory response. Polymer coating protects nucleic acid drug carriers from overexposure and has been shown to improve structural integrity and circulatory stability as well as to attenuate immune stimulation.

### Gene therapy

Gene therapy, as an indispensable tool in biomedical research, has shown potential to treat a variety of diseases, including single-gene inherited diseases, cancer, cardiovascular disease, diabetes, infectious diseases, and inflammatory diseases, which has profoundly influenced the development of medicine. Gene therapy is the treatment of diseases by introducing genetic material into cells and editing genes that produce defective proteins or interfering with gene expression^[58]^. Nucleic acids are the main tools of gene therapy, such as DNA and mRNA molecules for gene overexpression and small RNA molecules such as siRNA, miRNA, and antisense oligonucleotides for gene knockdown^[59]^. For example, Kusano *et al.*^[60]^ reported the potential therapeutic effect of intramuscular sonic hedgehog gene transfer on myocardial injury repair. In recent years, gene editing strategies based on the CRISPR/Cas9 system have been applied to the treatment of genetic diseases. The CRISPR/Cas9 system needs to guide nucleic acid sequence to control gene editing sites and is also a representative of nucleic acid participation in gene therapy^[61]^. The biggest limitation of gene therapy is the efficient delivery of gene regulatory systems to cells. Nucleic acid in its natural form is not easily absorbed by cells and is easily degraded and removed, so carriers are needed to deliver nucleic acid into cells. Although viral vectors such as adenoviruses, lentiviruses, and retrovirus show advantages in transfection rates and life-long expression, insertional mutations and other persistent side effects make clinical use difficult. EVs have nucleic acid and protein delivery functions and are potential gene therapy vectors.

### Immune regulation

Nucleic acid has the potential for immune regulation. In the process of biological evolution, higher organisms have evolved the mechanism of recognizing microbial nucleic acid sequences through pattern recognition receptors, thus activating anti-infection immunity^[62]^. This provides the basis for the immune regulation of nucleic acids. CpG oligodeoxynucleotide (CpG ODN) is a commonly used immune adjuvant that can effectively trigger a mammalian immune response through toll-like receptor 9 (TLR9) signaling and has been used as an immune adjuvant against infection and tumor^[63-70]^. In addition, poly I: C, PolyA: U, *etc.* may enhance the activity of nucleotide kinases and participate in immune regulation^[66,71]^.

In addition to the oligo nucleic acid chain, immune-gene therapy is another important way of nucleic acid immune regulation. It works by introducing genes that promote immune activation into the body’s cells. There are two cases. The first is the introduction and expression of cytokine genes to enhance the body’s immunity^[72,73]^. This method has broad spectrum and non-specificity. The other is a process that stimulates specific immunity by introducing specific epitope genes into the body. The method is also known as a nucleic acid vaccine (NAV). NAV aimes to introduce the gene sequence encoding specific antigen protein into animal somatic cells, synthesize antigen by using the protein expression system of animal itself, and induce the animal body to produce acquired immunity for the purpose of preventing and treating diseases. DNA vaccines are also known as naked vaccines, so named because they do not require any chemical vectors^[74]^. After the DNA vaccine is introduced into the host, it is taken up by cells (tissue cells, dendritic cells, or other antigen-presenting cells), and the antigen protein is expressed by using the protein synthesis system of the cells, which stimulates the host to produce cellular and humoral immunity through a series of cascading processes^[75,76]^. Compared with traditional inactivated vaccine, DNA vaccine has the following advantages: (1) enhanced immune protection; (2) sequence design can be used to modify antigen determinants or prepare polyvalent vaccines; and (3) producing a safe and durable immune response that does not require multiple immunizations. However, DNA vaccines are potentially dangerous: (1) Continuous expression of foreign antigens may have adverse consequences. Long-term expression of exogenous antigen by plasmids may lead to immune tolerance or anesthesia. (2) After being injected into the body, foreign DNA may be integrated into the host genome to inactivate or activate the tumor suppressor genes of the host cells and transform the host cells into cancer cells, which may be the worthiest of in-depth study among many safety issues of the nucleic acid vaccine.

mRNA vaccines can trigger a specific immune response by introducing mRNA encoding specific antigens into the body and using the protein synthesis mechanism of the host cell to produce antigens. Compared with traditional vaccines, mRNA vaccines are simpler to produce, faster to develop, do not require cell culture, have lower cost^[77]^, and are more immunogenic in expressing conformation stable proteins or exposing key antigen sites^[78-82]^. Even when compared with DNA vaccines, there are significant advantages. mRNA vaccines do not need to enter the nucleus, so they do not carry the risk of integration into the host genome^[83]^. However, two challenges must be overcome before mRNA vaccines work. The first challenge is the design and synthesis of mRNA. The high expression, specificity, and immunogenicity of kernel mRNA are important to the success of vaccines. In addition, mRNA also requires special design and modification to improve its stability. Another important challenge is the construction of a delivery carrier. A carrier with targeted properties can improve the enrichment of mRNA in the target cell, which is conducive to the efficient expression of the antigen. In this process, the lysosomal escape ability of the carrier is equally important for protecting mRNA from degradation. It is exciting that the approval of two coronavirus disease 2019 (COVID-19) mRNA vaccines (mRNA-1273 and BNT162b2) promote the development of mRNA vaccine technology. Clinical trials have shown that the two-dose regimen of BNT162b2 provides 95% protection against COVID-19 in humans over 16 years of age. Median safety over two months was same as other vaccines^[84]^. However, mRNA vaccines also have some problems to be solved, such as poor stability of the mRNA itself, low cell entry efficiency, and low translation efficiency^[85]^. The development of intracellular delivery carriers with nucleic acid protection has become a research focus in this field^[86-88]^.

## ENGINEERING STRATEGIES FOR NUCLEIC ACID-FUNCTIONALIZED EXTRACELLULAR VESICLES

Nucleic acid has developed into an important functional subassembly for the modification and functionalization of drug delivery carriers due to its unique physiological and biochemical properties. EVs, a rising star in drug delivery, has also sparkled with nucleic acid subassembly. Therefore, it is important to know the strategy of nucleic acid functionalization of EVs. The current engineering strategies of EVs with nucleic acid can be divided into two types: membrane modification and encapsulation. Each type contains several fabrication approaches. Commonly used fabrication approaches and their merits and demerits are summarized in Table 1.

**Table 1:** Comparison of different engineering strategies for nucleic acid-functionalized EVs

**Approach**	**Components**	**Merits**	**Demerits**	**Ref.**
Parental cell treatment	mRNA, SgRNA	High loading efficiency, no damage to EVs	The operation is difficult and the process complex	[89-91]
Incubation	siRNA	Facile method and easy to operate	Low load efficiency	[92]
Membrane modification	Aptamer, DNA hinge, CpG ODN, molecular beacon	Simple operation, high load efficiency	Nucleic acids are exposed to the surface and have no protective effect	[93-95]
Extrusion	siRNA	High load efficiency	Complex preprocessing	[96]
Electroporation	CpG ODN, siRNA, molecular beacon	Simple operation, high load efficiency	The formation of pores in EVs may cause irreversible damage	[94,97,98]
Sonication	miRNA, siRNA	Simple operation	Structural failure and low load efficiency for macromolecules	[99,100]
Streptolysin O	DNA junction, molecular beacon	Simple operation	The integrity of EVs may be impaired	[101,102]
Liposome	Plasmid, small RNA	Simple operation, high load efficiency	Particle size becomes larger, and EVs aggregates	[103,104]

EVs: Extracellular vesicles; CpG ODN: CpG oligodeoxynucleotides.

### Membrane modification strategies

**Figure 2:**
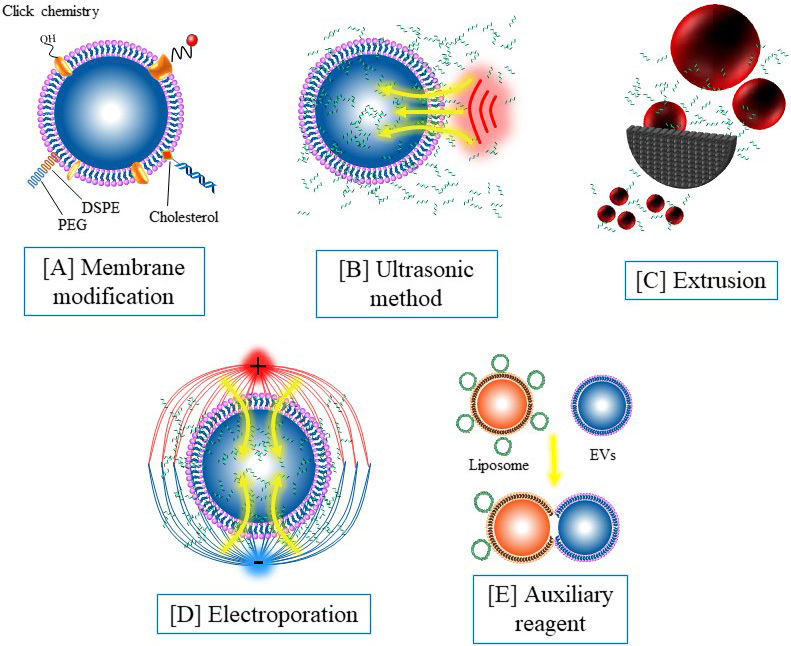
Engineering strategies for nucleic acid-functionalized extracellular vesicles: (A) membrane modification by using 1,2-distearoyl-sn-glycero-3-phosphorylethanolamine insertion, click chemistry, and covalent modification; (B) ultrasonic oscillations mediate nucleic acid loading; (C) nucleic acid loading mediated by extrusion; (D) nucleic acid loading mediated by electroporation; and (E) nucleic acid loading mediated by an auxiliary reagent.

By modifying specific chemical groups, the coupling between nucleic acid and EVs can be efficiently realized [Figure 2A]. Hydrophobic molecules such as 1,2-distearoyl-sn-glycero-3-phosphorylethanolamine (DSPE) can be inserted into the phospholipid bilayer of EVs. Nucleic acid molecules can be anchored to the surface of the vesicles by nucleic acid coupling DSPE^[93]^. Our previous study found that fresh EVs are rich in sulfhydryl groups, and nucleic acid can be conjugated with EVs by modifying the maleimide group with nucleic acid^[94]^. This method is mild and specific and has a wide application prospect. In recent years, click chemical modification based on glucose metabolism chemistry has been introduced into the engineering of EVs^[105-107]^, which also provides a promising approach for nucleic acid modification. The membrane modification strategies can anchor nucleic acid to the surface of the vesicles, thus endowing the EVs with targeted recognition and other functions. However, these loading methods leave the nucleic acid exposed to the outside of the EVs and cannot obtain the protection of the EVs.

### Encapsulation strategies

In addition to surface modifications, nucleic acid components can also be encased inside EVs [Figure 2B-E]. Parental cell treatment is an early method used to introduce nucleic acids into EVs. Although this method has high efficiency and simple follow-up operation, the preprocessing such as plasmid construction is still tedious and time-consuming. Electroporation is a transfection method that uses electrical pulses to create temporary holes in the plasma membrane to drive charged molecules in by establishing an electric potential in the membrane. Electroporation is an effective nucleic acid loading method and has been widely used in EVs for nucleic acid loading. In our previous work, molecular beacons were loaded into EVs through electroporation with a transfection efficiency at about 60%^[94]^. It has also been reported that incubation, extrusion, and sonication can induce nucleic acid to enter EVs. However, these methods are widely used in small molecule loading, but not widely used in nucleic acid loading due to their low efficiency for macromolecules. In recent years, the nucleic acid loading method using streptolysin O and liposome is a potential alternative to electroporation^[101]^. In contrast to membrane modification, encapsulation strategies can isolate nucleic acid from the external environment, avoiding premature exposure and degradation of nucleic acid.

## APPLICATION OF NUCLEIC ACID FUNCTIONALIZED EXTRACELLULAR VESICLES IN BIOMEDICINE

Nucleic acid-functionalized EVs have attracted extensive attention in biomedicine for their outstanding advantages. This section briefly highlights the current representative progress of nucleic acid-functionalized EVs to provide a preliminary understanding for interested researchers. The specific contents are summarized in Table 2.

**Table 2:** An overview of nucleic acid-functionalized EVs in nanomedicine.

**NA type**	**Function**	**EV origin**	**Loading strategy**	**Composition**	**Disease target**	**Outcomes**	**Ref.**
ncRNA	Regulate gene expression	MSCs	Parent cell treatment	Exogenous miR-let7c	Renal fibrosis	miR-let7c-MSC therapy attenuated kidney injury	[108]
	Regulate gene expression	HEK 293T	Parent cell treatment	circRNA	Depressive-like behaviors	Efficiently delivered circDYM to the brain and alleviated CUS-induced depressive-like behaviors	[109]
	Regulate gene expression	DCs	Electroporation	miR-let7	Breast cancer	Selectively targeted tumor tissues in tumor-bearing mice and inhibited tumor growth	[110]
	Regulate gene expression	MDA-MB231	Parent cell treatment	miRNA and siRNA	NA	Significantly reduced its therapeutic dose	[111]
siRNA	Interfering gene expression	Normal human fore-skin fibroblast	Electroporation	Alexa Fluor 647-tagged siRNA	KRAS^G12D^	Suppressed cancer in multiple mouse models of pancreatic cancer and significantly increased overall survival	[98]
	Interfering gene expression	Neuro2A cells or DCs	Co-incubation	Cholesterol-conjugated siRNAs	Human antigen R	Facilitated concentration-dependent silencing of human antigen R	[112]
	Interfering gene expression	Umbilical-cord-derived mesenchymal stem cells	Co-incubation	Hydrophobically modified siRNA	Huntingtin mRNA	Significant bilateral silencing of up to 35% of Huntingtin mRNA	[113]
	Interfering gene expression	HEK293T cells	Co-incubation	siRNA	Survivin gene	Significantly suppressed KB cell-derived cancer	[114]
	Interfering gene expression	HEK293T cells	Transfection	siRNA with 3WJ-folate arrow siRNA with 3WJ-cholesterol arrow	Survivin gene	Suppressed tumor growth in three animal models	[115]
mRNA	Coding protein	Mouse embryonic fibroblasts	Electroporation	mRNA	Phosphatase and tensin homologue-deficient glioma	RNA-containing exosomes restored tumor-suppressor function, enhanced inhibition of tumor growth, and increased survival	[116]
	Coding protein	HEK293	Parent cell treatment	mRNA	Cerebral ischemia	Reduced inflammation and promoted cell survival	[90]
DNA	Gene therapy	HEK293T cells	Transfection	Plasmid/DNA aptamers Pgc1α and Il-10 mRNA	PGC1α	Delivery of Pgc1α or Il-10 mRNA efficiently induced white adipocyte browning and alleviated IBD, respectively	[117]
	Immunotherapy	HEK293T cells	Transfection	Plasmid encoding Gag-OVA	CD4^+^ and CD8^+^ T-cell	Facilitated antigen cross-presentation and improved induced immunity	[118]
	Gene therapy	4T1 cells	Transfection	Minicircle DNA	TK-NTR	Mediated gene transfer that enables effective prodrug conversion and tumor cell death	[119]
	Drug delivery	RAW264.7	Electroporation	miR-21 molecular beacon	4T1	Realized a specific microRNA-responding delivery system for visual therapy of tumors	[94]
	Cancer target	RAW264.7	Membrane modification	AS1411 aptamer	MDA-MB231	Caused remarkable tumor tissue damage and reduced the percentage of proliferating Ki67-positive tumor cells	[93]
	Gene editing	SKOV3	Electroporation	DNA plasmid	SKOV3	Suppressed expression of poly(ADP-ribose) polymerase-1 (PARP-1), resulting in the induction of apoptosis in ovarian cancer	[91]
	Gene editing	HEK293FT	Liposome	DNA plasmid	MSCs	Endocytosed MSCs and expressed the encapsulated genes in the MSCs	[103]

EVs: Extracellular vesicles; MSCs: mesenchymal stem cells; DCs: dendritic cells.

### Nucleic acid-functionalized extracellular vesicles for targeted drug delivery

EVs have shown fascinating interest in the field of drug delivery and are regarded as promising for the next generation of nanomedicine. However, how to improve active targeting is an important problem for EVs. Aptamers can specifically recognize and bind to targets, showing great application potential in the construction of targeted drug delivery systems. Wan *et al.*^[93]^ reported targeting exosomes with aptamers carrying paclitaxel, a common anticancer drug in clinical practice. They covalently linked the AS1411 aptamer with cholesterol-PEG and subsequently grafted it onto mouse DC membranes. Then, modified DCs are mechanically extruded to create aptamer-guided nanovesicles. By using this extruding method, ~3 × 10^10^ targeted nanovesicles were obtained from approximately 1 × 10^7^ cells within 1 h. Chloe-PEG2000 was selected because of its amphiphilic and relatively rigid properties, which could stabilize nanovesicles by hydrophobic effect on the lipid bilayer. Strategies for preparing DSPE-aptamers may be used to mass produce targeted exosomes secreted by immune cells for cancer treatment. The approach is considered safer than cell-based immunotherapies because the vesicles have lost their ability to expand^[93]^.

Guo’s team reprogrammed exosomes using aptamer localization on the surface of exosomes to guide siRNA/miRNA cargo for targeted delivery and cancer treatment^[115]^. The authors designed a nanostructure with a three-way connection to make the ligands locate onto the interface of EVs. Placing membrane-anchored cholesterol at the tail of the three-way connection causes RNA aptamers or folic acid to appear on the outer surface of the EVs. Instead, placing cholesterol at the three-way arrow resulted in partial loading of RNA nanoparticles into vesicles. As a result, RNA nanostructures are directionally attached to the lipid bilayer membrane of EV, and the target ligand decorates the outer surface of EVs. This directionally engineered ligand showed that the engineered EVs can deliver siRNA to target cells specifically and realize effective blocking of tumor growth^[115]^. Recently, sgC8, an aptamer of membrane-bound protein tyrosine kinase 7, has been coupled to diacyl-lipids via PEG ligands in therapy platforms^[120]^. The immature dendritic cell-derived EVs are loaded with doxorubicin through electroporation, and then the EVs are functionalized by surface-targeting ligands through the hydrophobic effect^[120]^. This sgC8-guided exosome exhibits selective and dose-dependent cytotoxicity to human leukemia cells. In terms of the mechanism of cell internalization, studies have shown that clathrin-mediated endocytosis plays a major role in sgC8 aptamer-mediated endocytosis of various endocytosis pathways. These results suggest that targeted ligands themselves may influence exosome interactions with target cells^[120]^. Nevertheless, whether other ligand-target pairs affect EV internalization by different cancer cells remains to be determined.

### Nucleic acid-functionalized extracellular vesicles for gene therapy

Gene therapy is regarded as a possible cure to eradicate cancer and genetic diseases. The CRISPR/Cas9 system is a new gene editing tool and designed to work as a Cas9 nuclease single guide RNA (sgRNA) complex which has been widely used in life science. Recognizing the complementary 20-nucleotide genome sequence by sgRNA, Cas9 nuclease cleaves the double-stranded DNA and destroys three bases upstream of the adjacent motif of the target gene, leading to gene deletion, insertion, and mutation through error-prone non-homologous end linking or precise homologous directed repair. Although the CRISPR/Cas9 system is considered a promising gene therapy strategy, one key hurdle remains: the lack of a safe and effective way to transport the CRISPR/Cas9 system in the body. In recent years, EVs have been widely studied as promising drug delivery carriers, but their encapsulation efficiency of large nucleic acids is low. Lin *et al.*^[103]^ developed a hybrid method of exosomes and liposomes by simple incubation method. The synthesized hybrid nanoparticles effectively encapsulate the CRISPR-Cas9 plasmid, similar to liposomes. Further experiments showed that the synthesized hybrid nanoparticles could be incorporated into mesenchymal stem cells (MSCs) to express encapsulated genes that could not be transfected by liposomes alone. In another study, Kim *et al.*^[91]^ achieved tumor-targeted gene editing using tumor-derived EVs loaded with CRISPR/Cas9 plasmid by electroporation. These studies provide a new method for delivering the CRISPR/Cas9 system *in vivo*, which is expected to enable precise gene editing *in vivo* and be used in the treatment of cancer and other genetic diseases.

In addition to gene editing systems, gene therapy can also be achieved by regulating gene expression. Liu’s team^[95]^ used molecular beacons to silence the *miR-21* gene, thus enabling EV-mediated gene therapy. In earlier studies, the Kalluri group^[98]^ achieved targeted gene therapy for pancreatic cancer by using exosomes from normal fibroblast-like mesenchymal cells carrying interference sequences targeting oncogenic KrasG12D. Recently, the study entered a phase I clinical trial (ClinicalTrials.gov, Identifier: NCT03608631). Non-coding RNAs (ncRNAs) are natural tools for gene expression regulation and are also loaded into EVs for intracellular delivery and gene therapy^[108,109,111,121]^. Nucleic acid-functionalized EVs also show good application potential in tissue repair. Mathiyalagan *et al.*^[121]^ reported that EVs derived from CD34^+^ stem cells can target recipient cells and transfer miRNA precursors to regulate gene expression. In another study, Guo *et al.*^[122]^ used MSC-derived exosomes loaded with phosphatase and tensin siRNA for spinal injury repair. MSC-derived exosomes have been reported to have a protective effect in many diseases such as myocardial infarction^[9,123]^, bone defects^[124]^, and kidney diseases^[125]^ and can play a synergistic role with siRNA in tissue repair. These studies confirmed that EVs, as small RNA delivery carriers, have good potential in gene therapy. EVs have also been used to deliver large RNA. Yang *et al.*^[116]^ developed a technique for mass production of mRNA-encapsulating EVs through a homemade electroporation device. A new study found that both nerve growth factor mRNA and protein delivered via EVs can effectively treat ischemic brain injury^[90]^. This will further promote the application of nucleic acid-functionalized EVs in the biomedical field.

### Nucleic acid-functionalized extracellular vesicles used in immunotherapy

Immunotherapy has made remarkable achievements in clinical trials of malignant tumors, which brings new hope for tumor treatment. However, the suppressive state of the tumor immune microenvironment greatly limits the effect of immunotherapy. Therefore, regulating the immune state of the tumor microenvironment is of great significance to improve the effect of immunotherapy. CpG ODN can activate DCs and macrophages through TLR9, thus improving antigen presentation and immune activation effect. Yu *et al.*^[97]^ prepared exosomes from different origins and compared their physicochemical properties and delivery efficiency to verify whether EVs can effectively deliver immune-stimulating molecules to lymph nodes. It was found that EV encapsulation greatly increased the amount of internalization of immunomodulatory molecules, which induced higher tumor necrosis factor α (TNF-α) and interleukin-6 (IL-6) expression than free monophosphoryl lipid A (MPLA) and free CpG ODN. After subcutaneously loading CpG and MPLA exosomes, the expression of cytokines interferon-γ (IFN-γ) and TNF-α increased, and T cells were activated. This suggests that the delivery of immune adjuvants by extracellular vesicles is a potential immunotherapy strategy.

The nucleic acid vaccine is a new immunotherapy method. The intracellular delivery of nucleic acid and antigen expression can be effectively realized by loading the DNA or mRNA encoding antigen into EVs. In a preprint, Tsai *et al.*^[126]^ used exosome-mediated mRNA delivery as a severe acute respiratory syndrome coronavirus (2SARS-CoV-2) vaccine. The results show that the vaccine triggered long-term antiviral immune responses include cellular and humoral immunity, suggesting that exosome-based mRNA formulations represent a previously untapped platform for combating coronavirus disease 2019 (COVID-19). Recently, Allele Biotechnology and Pharmaceutical^[75]^ announced that they have designed an induced pluripotent stem cell (iPSC) line carrying genes encoding multiple SARS-COV-2 antigen. This iPSC line can release large amounts of EVs that carry viral mRNA and proteins. Alleles indicated that the engineered cell line conquers two problems: (1) vaccines containing multiple antigens may have better performance than vaccines containing single mRNAs, such as Pfizer/biotech and Moderna vaccines; and (2) while the Pfizer/BioNTech vaccines need to be stored at -80 °C, iPSC-derived EVs prevent messenger RNA degradation, making RNA remain intact for several months at 4 °C.

## CHALLENGES AND PROSPECTS

Nucleic acid-functionalized EVs show great application prospects in the biomedical field. It enables EVs to be a promising candidate in the hot areas of targeted drug delivery, gene therapy, and immunotherapy. However, some challenges to using nucleic acid-functionalized EVs remain. Firstly, the lack of research methods on EVs has greatly hindered the development of nucleic acid-functionalized vesicles. The low natural production rate of EVs greatly affects mass production. At the same time, EVs are heterogeneous, and it is difficult to obtain high purity homogeneity subgroups by existing isolation techniques. Although purified EVs can be isolated from cell lines secreting EVs, these EVs have immunogenic and carcinogenic potential. This greatly impedes downstream modification, quality evaluation, and clinical application. Secondly, RNA-based nucleic acid functionalization is affected by the lack of stability of RNA, which is easily destroyed and leads to the failure of functionalization. In addition to the above outstanding problems, nucleic acid-functionalized EVs are also faced with the lack of modification methods, the dilemma of selection of EVs, and the difficulty of clinical transformation. Nevertheless, nucleic acid-functionalized EVs provide a new tool for biomedicine with great potential and application prospects.

Reviewing the latest research progress, we speculate that nucleic acid-functionalized EVs will become a hot research area in the future. We boldly forecast its future research direction. The multi-functional diagnosis and treatment platform based on the programmable characteristics of the nucleic acid will realize personalized and precise treatment. Nucleic acid has programmable performance and can achieve intelligence and multi-function through sequence design. Nucleic acid-functionalized EVs enable the EVs to acquire intelligent characteristics such as stimulus response, intelligent controlled release, and therapeutic feedback. It promises to provide new strategies for personalization and precision medicine. A gene-editing system based on EVs is expected to achieve precise and efficient gene therapy. CRISPR, a gene-editing system, has made significant progress at the cellular level, showing satisfactory gene editing efficiency. However, *in vivo* gene editing is still hampered by the lack of delivery vectors. EVs are natural delivery carriers of bioactive molecules and have the ability to allow bioactive molecules to escape from lysosomes. Recent studies have found that EVs have tissue targeting ability such as homologous targeting. A gene-editing system developed by EVs is expected to achieve accurate and efficient gene editing *in vivo*.

## DECLARATIONS

### Copyright

© The Author(s) 2022.
